# The Use of Bio-Guided Fractionation to Explore the Use of Leftover Biomass in Dutch Flower Bulb Production as Allelochemicals against Weeds

**DOI:** 10.3390/molecules18044510

**Published:** 2013-04-17

**Authors:** Dinar S. C. Wahyuni, Frank van der Kooy, Peter G. L. Klinkhamer, Rob Verpoorte, Kirsten Leiss

**Affiliations:** 1 Natural Product Laboratory, Institute of Biology, Leiden University, 2300 RA Leiden, The Netherlands; E-Mails: f.vanderkooy@uws.edu.au (F.K.); verpoort@chem.leidenuniv.nl (R.V.); 2 Biology Department, Faculty of Mathematics and Sciences, Sebelas Maret University, Surakarta 57126, Indonesia; 3 Plant Ecology and Phytochemistry Department, Institute of Biology, Leiden University, 2300 RA Leiden, The Netherlands; E-Mails: p.g.l.klinkhamer@biology.leidenuniv.nl (P.G.L.K.); k.a.leiss@biology.leidenuniv.nl (K.L.); 4 Centre for Complementary Medicine Research, University of Western Sydney, Locke Bag 1797, Penrith South DC, NSW 1797, Australia

**Keywords:** allelochemicals, bio-guided fractionation, flower bulbs, alkaloids, weeds

## Abstract

A major problem in flower bulb cultivation is weed control. Synthetic herbicides are mainly used, although they cause a range of problems, and integrated weed control through application of naturally occurring allelochemicals would be highly desirable. Flower bulb production creates large amounts of leftover biomass. Utilizing this source for weed control may provide new applications of the bulb crops. We therefore screened 33 flower bulb extracts for allelochemical activity against weeds. Several methanol and chloroform extracts were observed to inhibit germination and growth of *Senecio vulgaris* L. and *Lolium perenne* L., as representatives of di- and mono-cotyledonous weeds, respectively. Narciclasine was identified as the bioactive compound in *Narcissus*. The extract of *Amaryllis belladonna* L. was equally active, but did not contain any narciclasine. Bioassay-guided fractionation of the *A. belladonna* extract resulted in the identification of lycorine as the bio-active compound. The IC_50_ measured for radicle growth inhibition was 0.10 µM for narciclasine and 0.93 µM for lycorine, compared to 0.11 mM of chlorpropham, a synthetic herbicide. Therefore, the leftover biomass from the spring bulb industry represents an interesting potential source for promising allelochemicals for further studies on weed growth inhibition.

## 1. Introduction

Worldwide the economic value of spring flower bulb production is estimated to be over $1 billion. With 21,000 ha of tulip, lily, narcissus, gladiolus, hyacinthns, crocus and iris production, The Netherlands alone are by far the major global producer, contributing some $756 million in value [1]. 

A major problem in bulb production is weed control. Bulbs are generally poor competitors due to their limited root system and their relatively open canopy [2]. In addition, due to the relatively long growing season, both winter and summer weeds need to be controlled. Weed infestations have been shown to substantially reduce total bulb yield and weight, as well as number of flowers produced [3,4]. Due to fewer flowers and smaller bulbs the effect of weed competition may even carry over into the following crop season [5]. Additionally, the presence of weeds can severely obstruct the harvesting of the bulb crop [6].

Presently the use of synthetic herbicides is the method of choice for weed control in flower bulb cultivation [2]. However, these chemicals can cause negative side effects for the bulb crop. When applied in mid-summer to control summer weeds, they can reduce bulb size and flower number in the subsequent spring [7]. More generally the widespread and excessive use of herbicides has led to a build-up of herbicide resistance [8] and has raised concerns about negative impacts on human health, non-target beneficial organisms and the environment [9]. At the same time adaptation of new European Union regulations regarding pesticide registration and application results in a considerable reduction of the options for pesticide application [10]. Therefore, integrated weed management for bulb production using an array of different complementary control tactics seems the way forward [1,11]. An important novelty in such an approach is the use of natural weed control products [12,13].

Already more than 200,000 secondary plant compounds have been identified, all in one way or another involved in the interaction with other plants or organisms [3]. This provides an immense source for the generation of natural crop protectants. Allelochemicals comprise secondary plant compounds affecting the growth of other plants. Allelochemicals suppressing or eliminating competing plant species are interesting sources of natural weed protectants [14]. Up to now there are only a handful of examples of natural products used for weed management [13]. Allelopathic bioassays applied in phytochemical studies evaluate the bioactivity of plant extracts by measuring inhibition of germination and seedling growth [15]. Promising extracts are subsequently subjected to bio-guided fractionation to identify the bio-active compound [16].

Recently, the interest in alternative uses of agricultural crops, including, flower bulbs, has grown in The Netherlands. This is based on the Dutch government stimulating a bio-based economy using crops and leftover biomass from the agricultural sector for non-food industrial purposes [17]. The production of flower bulbs creates relatively large amounts of leftover biomass in the form of stems, leaves and flowers as well as small bulbs which are unsuitable for production. However, these leftovers might be a source for other novel high value products, such as medicines, flavors, fragrances, dyes and crop protectants. We, therefore, explored the potential of leftover biomass from flower bulb production as a potential source of allelochemical weed protectants for further study.

Consequently, in this study the effect of 33 extracts of bulb plants on weed germination and growth inhibition was bioassayed using *Senecio vulgaris* L. (common groundsel) and *Lolium perenne* L. (perennial ryegrass) as representatives of di- and monocotyledons, respectively. Bio-guided fractionation was subsequently used to identify and quantify the bio-active compounds in the most active extracts.

## 2. Results and Discussion

The screening of the flower bulb extracts resulted in the identification of potential sources for natural products against weeds. Six methanol extracts of the 33 flower bulb species showed significant inhibition of both *L. perenne* (Table 1) and *S. vulgaris* (Table 2) seed germination at concentrations of 1 and 0.5 mg/mL. In addition, one chloroform extract was active at both concentrations against germination of *L. perenne*. Inhibition of *S. vulgaris* was further observed with two chloroform extracts at both concentrations, one chloroform extract at 1 mg/mL and four methanol extracts at 1 mg/mL. *Narcissus pseudonarcissus* L. cv. Carlton was the only flower bulb species inhibiting both weed species with methanol and chloroform extracts at both concentrations tested. Complete inhibition of germination in *L. perenne*, at both concentrations, was only achieved by the chloroform extract of *N. pseudonarcissus*. In *S. vulgaris*, germination was completely suppressed by *N. pseudonarcissus* cv. Carlton and *Narcissus tazetta* L. cv. Grand soleil d’or with methanol and chloroform extracts at both concentrations. Also *Narcissus tazetta* L. cv. Avalanche, *Narcissus poeticus* L. cv. Recurvus, *Hippeastrum* Herb. Hybrid cv. Misty and *Hippeastrum cybister* Benth. & Hook. f. cv. Chico at 1 mg/mL caused 100% germination inhibition in *S. vulgaris*. All active extracts belonged to the *Amaryllidaceae*.

**Figure 1:**
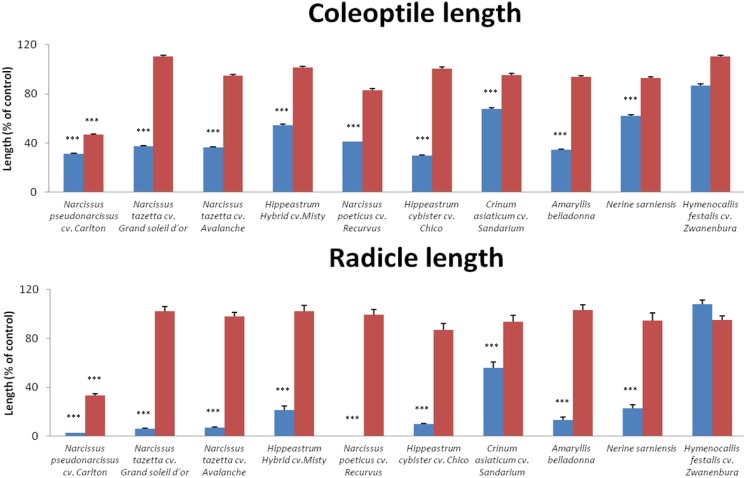
The effect of ten active methanol extracts on coleoptile and radical lengths of *Senecio vulgaris* seeds at 0.1 and 0.01 mg/mL. Data present means and standard deviations of 3 replicates. Significant differences between control and treatment are indicated as: *** *p* ≤ 0.001.

Based on these results, six methanol extracts active against both weeds at both concentrations, including *N. pseudonarcissus* cv. Carlton, *N. tazetta* cv. Grand soleil d’or, *N. tazetta* cv. Avalanche, *N. poeticus* cv. Recurvus, *H. *Hybrid cv. Misty and *H. cybister* var. Chico, as well as the four methanol extracts (*Crinum asiaticum* L. cv. Sandarium, *Amaryllis belladonna* L., *Nerine sarniensis* Herb. and *Hymenocallis festalis* Hort. ex Schmarse cv. Zwanenburg) active against *S. vulgaris* at the higher concentration of 1 mg/mL, were selected for a subsequent growth inhibition bioassay on *S. vulgaris* at concentrations of 0.1 and 0.01 mg/mL. All these extracts significantly inhibited coleoptile and radicle growth of *S. vulgaris* (F = 371.933, df = 10, *p* ≤ 0.000 and F = 411.185, df = 10, *p* ≤ 0.000, respectively) (Figure 1), except *H. festalis* cv. Zwanenburg, which only affected coleoptile growth. In general, radicle growth was more inhibited compared to coleoptile growth. The extracts of the *Narcissus* sp. caused almost complete inhibition of radicle growth.

**Table 1:** The effect of methanol and chloroform extracts, derived from 33 flower bulb species, on *Lolium perenne* seed germination at concentrations of 0.5 and 1.0 mg/mL. Data present the mean of three replicates. Significant inhibition is indicated as: *** *p* ≤ 0.001.

No.	Scientific Name	Family	Part of plant	Germination inhibition of *L. perenne*(%)			
				Methanol Extracts		Chloroform Extracts	
				1 mg/mL	0.5 mg/mL	1 mg/mL	0.5 mg/mL
1.	*Narcissus pseudonarcissus* cv. Carlton	Amaryllidaceae	Bulb	96.7 ***	96.7 ***	100.0 ***	100.0 ***
2.	*Narcissus tazetta* cv. Grand soleil d’or	Amaryllidaceae	Bulb	86.7 ***	56.7 ***	16.7	16.7
3.	*Narcissus tazetta* cv. Avalanche	Amaryllidaceae	Bulb	86.7 ***	53.3 ***	23.3	6.7
4.	*Hippeastrum *Hybrid cv. Misty	Amaryllidaceae	Bulb	76.7 ***	90.0 ***	6.7	10.0
5.	*Narcissus poeticus *cv. Recurvus	Amaryllidaceae	Bulb	99.7 ***	86.7 ***	20.0	3.3
6.	*Hippeastrum cybister *cv. Chico	Amaryllidaceae	Bulb	86.7 ***	53.3 ***	3.3	16.7
7.	*Crinum asiaticum *cv. Sandarium	Amaryllidaceae	Bulb	20.0	3.3	93.3 ***	30.0
8.	*Amaryllis belladonna*	Amaryllidaceae	Bulb	30.0	10.0	00.0	3.3
9.	*Nerine sarniensis*	Amaryllidaceae	Bulb	26.7	16.7	3.3	3.3
10.	*Hymenocallis festalis *cv. Zwanenburg	Amaryllidaceae	Leaf	10.0	10.0	6.7	10.0
11.	*Gladiolus* cv. Rose Supreme	Iridaceae	Flower	20.0	6.7	3.3	6.7
12.	*Arisaema* cv. Tortuosum	Araceae	Bulb	20.0	6.7	3.3	3.3
13.	*Anemone* *Coronaria* cv. De Caen	Ranunculaceae	Leaf	6.7	6.7	16.7	13.3
14.	*Allium spherocephalon*	Amaryllidaceae	Flower	6.7	3.3	16.7	3.3
15.	*Oxalis triangularis*	Oxalidaceae	Flower	13.3	10.0	10.0	6.7
16.	*Scilla siberica *cv. Spring Beauty	Asparagaceae	Flower	3.3	10.0	3.3	13.3
17.	*Ornithogalum thyrsoides* cv. Mount Fuji	Asparagaceae	Leaf and stem	6.7	20.0	10.0	10.0
18.	*Hyacinthus orientalis *cv. White Pearl	Asparagaceae	Leaf	10.0	3.3	6.7	00.0
19.	*Babiana stricta*	Iridaceae	Leaf	10.0	6.7	3.3	00.0
20.	*Amarine tubergenii* cv. Zwanenburg	Asparagaceae	Flower	16.7	16.7	10.0	3.3
21.	*Zephyranthes robusta*	Amaryllidaceae	Flower	10.0	10.0	0.0	0.0
22.	*Ixia* *paniculata* cv. Yellow Emperor	Iridaceae	Flower	10.0	6.7	6.7	30.0
23.	*Caladium* cv. Postman Joyner	Araceae	Bulb	3.3	3.3	6.7	3.3
24.	*Begonia* cv. Compacta	Begoniaceae	Bulb	3.3	3.3	6.7	6.7
25.	*Dahlia* cv. Deca Split Pink Bells	Iridaceae	Bulb	3.3	10.0	13.3	3.3
26.	*Eucomis comosa*	Liliaceae	Bulb	3.3	13.3	6.7	6.7
27.	Iris X hollandica cv. Apollo	Iridaceae	Bulb	10.0	3.3	3.3	3.3
28.	*Incarvillea delavayi*	Bignoniaceae	Leaf	13.3	10.0	3.3	13.3
29.	*Arisaema galeatum*	Araceae	Leaf	6.7	10.0	6.7	6.7
30.	*Dahlia* cv. Apricot Star	Iridaceae	Leaf	3.3	16.7	13.3	13.3
31.	*Lilium candidum*	Liliaceae	Bulb	10.0	13.3	13.3	3.3
32.	*Hyacinthus orientalis* cv. Delft Blue	Asparagaceae	Bulb	3.3	10.0	6.7	16.7
33.	*Fritallaria*	Liliaceae	Bulb	10.0	3.3	0.0	0.0

**Table 2:** The effect of methanol and chloroform extracts, derived from 33 flower bulb species, on *Senecio vulgaris* seed germination at concentrations of 0.5 and 1.0 mg/mL. Data present the mean of three replicates. Significant inhibition is indicated as: *** *p* ≤ 0.001 and ** *p* ≤ 0.01.

No.	Scientific Name	Family	Part of plant	Germination inhibition of *S. vulgaris *(%)			
				Methanol Extracts		Chloroform Extracts	
				1 mg/mL	0.5 mg/mL	1 mg/mL	0.5 mg/mL
1.	*Narcissus pseudonarcissus* cv. Carlton	Amaryllidaceae	Bulb	100.0 ***	100.0 ***	100.0 ***	100.0 ***
2.	*Narcissus tazetta* cv. Grand soleil d’or	Amaryllidaceae	Bulb	100.0 ***	100.0 ***	100.0 ***	100.0 ***
3.	*Narcissus tazetta* cv. Avalanche	Amaryllidaceae	Bulb	100.0 ***	100.0 ***	100.0 ***	16.7 ***
4.	*Hippeastrum *Hybrid cv. Misty	Amaryllidaceae	Bulb	100.0 ***	96.7 ***	0.0	0.0
5.	*Narcissus poeticus *cv. Recurvus	Amaryllidaceae	Bulb	100.0 ***	90.0 ***	3.3	0.0
6.	*Hippeastrum cybister *cv. Chico	Amaryllidaceae	Bulb	100.0 ***	86.7 ***	0.0	0.0
7.	*Crinum asiaticum *cv. Sandarium	Amaryllidaceae	Bulb	93.3 ***	30.0	0.0	0.0
8.	*Amaryllis belladonna*	Amaryllidaceae	Bulb	90.0 ***	6.7	6.7	0.0
9.	*Nerine sarniensis*	Amaryllidaceae	Bulb	83.3 ***	0.0	16.7	0.0
10.	*Hymenocallis festalis *cv. Zwanenburg	Amaryllidaceae	Leaf	43.3 **	0.0	0.0	0.0
11.	*Gladiolus* cv. Rose Supreme	Iridaceae	Flower	0.0	0.0	0.0	0.0
12.	*Arisaema* cv.Tortuosum	Araceae	Bulb	0.0	3.3	0.0	0.0
13.	*Anemone* *Coronaria* cv. De Caen	Ranunculaceae	Leave	0.0	0.0	0.0	0.0
14.	*Allium spherocephalon*	Amaryllidaceae	Flower	0.0	0.0	3.3	0.0
15.	*Oxalis triangularis*	Oxalidaceae	Flower	0.0	0.0	0.0	0.0
16.	*Scilla siberica *cv. Spring Beauty	Asparagaceae	Flower	0.0	0.0	0.0	0.0
17.	*Ornithogalum thyrsoides* cv. Mount Fuji	Asparagaceae	Leaf and stem	0.0	0.0	0.0	0.0
18.	*Hyacinthus orientalis *cv. White Pearl	Asparagaceae	Leaf	0.0	0.0	0.0	0.0
19.	*Babiana stricta*	Iridaceae	Leaf	0.0	0.0	0.0	0.0
20.	*Amarine tubergenii* cv. Zwanenburg	Asparagaceae	Flower	0.0	0.0	0.0	0.0
21.	*Zephyranthes robusta*	Amaryllidaceae	Flower	0.0	0.0	0.0	0.0
22.	*Ixia* *paniculata* cv.Yellow Emperor	Iridaceae	Flower	0.0	0.0	0.0	0.0
23.	*Caladium* cv. Postman Joyner	Araceae	Bulb	0.0	0.0	0.0	0.0
24.	*Begonia* cv. Compacta	Begoniaceae	Bulb	0.0	0.0	0.0	0.0
25.	*Dahlia* cv. Deca Split Pink Bells	Iridaceae	Bulb	0.0	0.0	0.0	0.0
26.	*Eucomis comosa*	Liliaceae	Bulb	0.0	0.0	0.0	0.0
27.	Iris X hollandica cv. Apollo	Iridaceae	Bulb	0.0	0.0	0.0	0.0
28.	*Incarvillea delavayi*	Bignoniaceae	Leaf	0.0	0.0	0.0	0.0
29.	*Arisaema galeatum*	Araceae	Leaf	0.0	0.0	0.0	0.0
30.	*Dahlia* cv. Apricot Star	Iridaceae	Leaf	0.0	0.0	0.0	0.0
31.	*Lilium candidum*	Liliaceae	Bulb	0.0	0.0	0.0	0.0
32.	*Hyacinthus orientalis* cv. Delft Blue	Asparagaceae	Bulb	0.0	0.0	0.0	0.0
33.	*Fritallaria*	Liliaceae	Bulb	0.0	0.0	0.0	0.0

**Figure 2:**
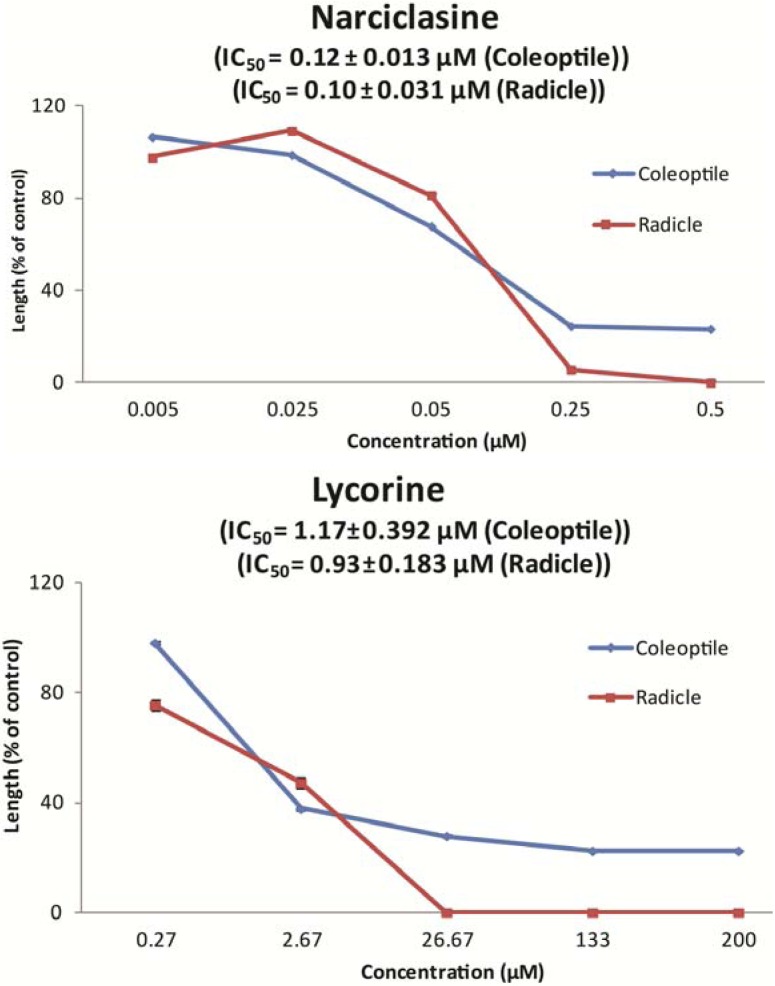
Dose-response curves of growth inhibition of *Senecio vulgaris* seedlings treated with narciclasine and lycorine. Data present the mean and standard deviation of three replicates. Where not visible, bars fall within symbols.

Subsequently, the 10 methanol extracts tested were subjected to high-performance liquid chromatography (HPLC) and liquid chromatography-mass spectrometry (LC-MS) analysis for identification of the active compounds inhibiting germination. Based on UV spectra (λ_max_ = 330, 305, 252 nm), mass spectra (MS: *m/z* 307 (M^+^)) and comparison with the standard compound, the active compound was identified as narciclasine. All *Narcissus* species, except *N. tazetta* cv. Grand soleil d’or, contained narciclasine, attaining a maximum in *N. pseudonarcissus* cv. Carlton with 0.25% of the bulb fresh weight (Table 3). To confirm that narciclasine is a germination inhibitor of *S. vulgaris* we conducted an inhibition bioassay measurement with narciclasine at five concentrations in the range of 0.005 to 0.5 µM. The concentration at which 50% of the seedling coleoptile growth was inhibited (IC_50_) was 0.12 ± 0.013 µM and that of the radicle growth 0.10 ± 0.031 µM. (Figure 2). Complete inhibition of radicle growth was obtained at a concentration of 0.5 µM. All *Narcissus* sp. included in this study contained much higher concentrations of narciclasine than 0.5 µM; particularly *N. pseudonarcissus* with a concentration of 0.88 µM was rich in narciclasine. In comparison, the commercial herbicide chlorpropham, used as positive control for our bioassays, has an IC_50_ of 0.20 mM for inhibition of coleoptile and 0.11 mM for radicle growth. Narciclasine is thus a much more potent growth inhibitor.

**Table 3:** Concentration of narciclasine and lycorine in ten flower bulb methanol extracts inhibiting germination of *Senecio vulgaris* seeds.

No.	Plant name	Narciclasine		Lycorine	
		µM	mg/kgW	µM	mg/kgW
1.	*Narcissus pseudonarcissus *cv. Carlton	0.88 ^a^	0.27 ^a^	nd	nd
2.	*Narcissus tazetta* cv. Grand soleil d’or	nd	nd	8.58 ^a^	2.47 ^a^
3.	*Narcissus tazetta* cv. Avalanche	0.20 ^a^	0.06 ^a^	5.22 ^a^	1.50 ^a^
4.	*Hippeastrum *Hybrid cv. Misty	nd	nd	3.26 ^b^	0.94 ^b^
5.	*Narcissus poeticus *cv. Recurvus	0.07 ^b^	0.02 ^b^	2.14 ^b^	0.61 ^b^
6.	*Hippeastrum cybister *cv. Chico	nd	nd	nd	nd
7.	*Crinum asiaticum *cv. Sandarium	nd	nd	5.36 ^b^	1.54 ^b^
8.	*Amaryllis belladonna*	nd	nd	23.21 ^b^	6.67 ^b^
9.	*Nerine sarniensis*	nd	nd	29.43 ^b^	8.45 ^b^
10.	*Hymenocallis festalis *cv. Zwanenburg	nd	nd	nd	nd

a: in fresh bulbs; b: in dried bulbs; nd: not detected.

Narciclasine is an isocarbostyril alkaloid, first isolated from *Narcissus* bulbs [18]. An average amount of 100 mg/kg in *Narcissus* Carlton bulbs has been reported [18,19], however we detected only 0.27 mg/kg of narciclasine in this cultivar. Similarly, Lubbe *et al.* [20] reported amounts of about 0.5 mg/kg of narciclasine in *Narcissus* cv. Carlton from The Netherlands, as determined by ^1^H-NMR spectroscopy. Piozzi *et al.* [19] observed that the contents of narciclasine in *Narcissus incomparabilis* Mill. bulbs was 50% lower at the end of the season. We do not know at which stage the bulbs used for our extracts were harvested, but Lubbe *et al.* [20], studying the accumulation of major alkaloids in *Narcissus* over the season, did not report any seasonal changes in the amount of narciclasine in *Narcissus* Carlton, so possibly the difference in the amount of narciclasine may be explained by bulb origin [18]. Both Carlton cultivars with relative low amounts originated from The Netherlands, while the origin of the Carlton bulbs with the relatively high amounts is unknown. Differences in alkaloid contents in *Hymenocallis littoralis* Salisb. from different origins, of the magnitude observed in our study, have been reported by Pettit *et al.* [21], who stated that bulbs from a warmer and drier source produce significantly more alkaloids. Although narciclasine also occurs in some other species of the *Amaryllidaceae*, *Narcissus* bulbs remain the best and easiest to obtain narciclasine [22]. Narciclasine is already known as a potent plant growth inhibitor. Concentrations of 0.25 µM inhibited 50% of wheat grain radicle growth, while 1.25 µM led to almost complete inhibition [18]. In comparison with our results, 0.12 µM inhibited 50% of *S. vulgaris* coleoptile and radicle growth, while 0.5 µM caused strong inhibition of coleoptile growth and complete inhibition of radicle growth. Narciclasine is thus a much more potent growth inhibitor. Narciclasine isolated from mucilage of *Narcissus tazetta* bulbs, exhibited a wide range of inhibitory effects [23]. Seed germination in rice and Chinese cabbage was reduced to 5% in the presence of 10 µM narciclasine, while radicle growth was strongly reduced and the growth of coleoptiles completely inhibited. In comparison with our data, a concentration of 0.25 µM led to 5% radicle growth and 25% of coleoptile growth of *S. vulgaris*. The effect we observed for narciclasine was much more potent for *S. vulgaris*. Narciclasine also inhibited accumulation of chlorophyll and chlorophyll proteins [23]. Research on the mode of action of narciclasine is limited, although it is known that it inhibits protein synthesis [24] and exhibits antimitotic activity [18]. Recently, Hu *et al.* [25] showed that narciclasine inhibits the auxin signaling pathway in *Arabidopsis* roots. As such, narciclasine appears to be a general growth inhibitor with an effect on a rather broad range of plants. Up to now narciclasine has not been used as a growth inhibitor in agri- and horticulture, while it is intensively investigated for its medicinal properties [26], especially for its selective anti-cancer effects [22,27].

**Figure 3:**
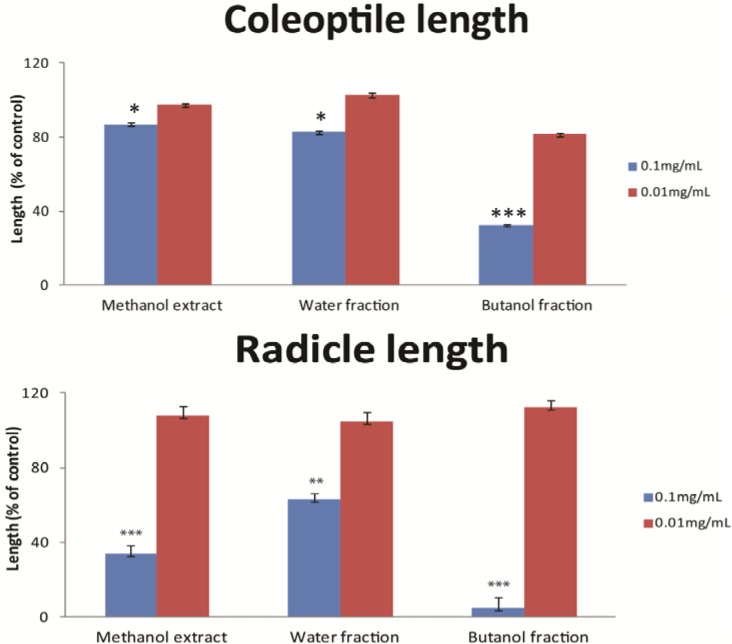
The effect of water and butanol fractions of a methanol extract of *Amaryllis belladonna* on coleoptile and radicle length of *Senecio vulgaris* seeds at concentrations of 0.1 and 0.01mg/mL. Effects are expressed as coleoptile or radicle length of the treatment as a percentage of the corresponding length of coleoptile or radicle length of the control. Data present means and standard deviations of three replicates. Significant differences between control and treatment are indicated as * *p* ≤ 0.05, ** *p* ≤ 0.01 and *** *p* ≤ 0.001.

Besides *N. tazetta* cv. Grand soleil d’or, the other two extracts which inhibited weed germination, but according to the LC-MS analysis did not contain any narciclasine, were *H. cybister* cv. Chico and *A. belladonna*. To identify the active compounds in these extracts we applied bio-guided fractionation. To obtain sufficient material for extraction we chose *A. belladonna* which has a relatively big bulb size. The original methanol extract was separated into four fractions by liquid-liquid partioning with water, hexane, chloroform and butanol. Only the water and butanol fractions provided sufficient volumes to conduct germination inhibition bioassays. All fractions significantly inhibited coleoptile and radicle growth of *S. vulgaris* at 0.1 mg/mL, while no activity was observed at 0.01 mg/mL (Figure 3). The butanol fraction at 0.1 mg/mL was most active resulting in 77% inhibition of the coleoptile growth (F = 39.503, df = 2, *p* ≤ 0.000) compared to the control and almost inhibited radicle growth completely (F = 68.128, df = 2, *p* ≤ 0.000). We, therefore, partitioned the butanol fraction further into nine different fractions using a Sephadex LH-20 chromatography column.

**Figure 4:**
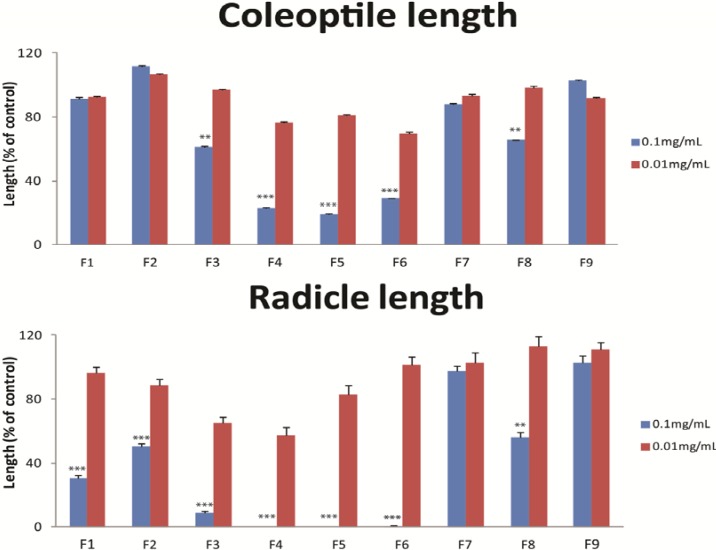
The effect on nine sub-fractions of a butanol fraction, derived from a methanol extract of *Amaryllis belladonna*, on coleoptile and radicle length of *Senecio vulgaris* seeds at concentrations of 0.1 and 0.01 mg/mL. Effects are expressed as coleoptile or radicle length of the treatment as a percentage of the corresponding length of coleoptile or radicle length of the control. Data present means and standard deviations of three replicates. Significant differences between control and treatment are indicated as ** *p* ≤ 0.01 and *** *p* ≤ 0.001.

These fractions were subsequently tested in the germination inhibition assay at concentrations of 0.1 and 0.01 mg/mL. Fractions 3, 4, 5 and 8 showed a significant inhibition of coleoptile growth (F = 276.666, df = 9, *p* ≤ 0.000), while fractions 1, 2, 3, 4, 5, 6 and 8 significantly inhibited radicle growth (F = 299.146, df = 9, p ≤ 0.000, Figure 4). Inhibition of coleoptile and radicle growth, with almost complete radicle inhibition, was strongest in fractions 4, 5 and 6. Subsequently, these three fractions were subjected to HPLC and LC-MS analysis for identification of the active compounds. Based on UV spectra [λ_max_ = 292, 236 (sh), 206 nm], mass spectra [MS: *m/z* 288 (M^+^)] and comparison with a standard, the active compound was identified as lycorine. Quantification of lycorine showed that *N. sarniensis* and *A. belladonna* contained relatively high amounts of lycorine, comprising 6.67 mg/kg and 8.45 mg/kg of the bulbs dry weight, respectively (Table 3). Furthermore, all *Narcissus* sp., except *N. pseudonarcissus*, as well as *C. asiaticum* contained lycorine. To confirm that lycorine is a germination inhibitor of *S. vulgaris* we conducted an inhibition bioassay with lycorine using 5 concentrations in the range of 0.3 to 200 µM. The concentration at which 50% of the seedling coleoptile growth was inhibited (IC_50_) was 1.17 ± 0.192 µM and that of the radicle growth 0.93 ± 0.183 µM. (Figure 2). Complete inhibition of radicles was obtained at a concentration of 30 µM of lycorine. In all those flower bulb species containing lycorine the concentration of lycorine was much higher than 30 µM. Particularly bulbs of *A. belladonna* containing 23.21 µM and *N. sarniensis* containing 29.43 µM were rich in lycorine and thus form a promising natural source for this compound. Lycorine is a much more potent growth inhibitor compared to chlorpropham with an IC_50_ of the coleoptile of 0.20 mM and of radicle growth of 0.11 mM.

Lycorine is an isocarbostyril alkaloid and was the first alkaloid detected in the *Amaryllidaceae* [28]. Within this plant family it is reported to occur in several genera, including *Amaryllis*, *Crinum* and *Narcissus* [26], as well as *Hippeastrum* [29,30,31,32,33] and *Nerine* [34]. Within the *Narcissus* genus lycorine occurs in *N. tazetta* [26], which was confirmed by our results. The highest amount of lycorine in our study was observed in *A. belladonna*: 8 mg/kg (dry weight). In contrast, 75 mg/kg (dry weight) of lycorine was reported to be present in *A. belladonna* bulbs grown in Australia [35]. The *A. belladonna* bulbs we used for our bulk extract came from The Netherlands, which compared to Australia is much colder and wetter. Indeed, the amount of lycoridine, a derivative from lycorine, in *H. littoralis* from Arizona, with a hot and dry climate, amounted to 118 mg/kg, while only 15 mg/kg were measured in the same bulb species originating from Hawaii [21]. Lycorine has also been described as a potent growth inhibitor. Hindrance of plant growth by lycorine is caused by its inhibitory effect on ascorbic acid biosynthesis [36]. Concentrations of 10^−6^ mol/L inhibited cell division in pea and oat plants [37]. Studying the allelopathic effect of L. *Lycoris radiata* Herb. bulbs [38] and leaves [14] lycorine was identified as the active compound inhibiting plant growth. In the later study a concentration of 0.85 mg/mL of an aqueous leaf extract completely inhibited the growth of seedling radicles and hypocotyls in alfalfa, Chinese cabbage, timothy and lettuce. Based on these results, the use of *L. radiata* plants as ground cover and the use of their dead leaves as a cover mulch to control weeds was proposed.

In the present study we identified narciclasine and lycorine as bioactive substances in spring flower bulbs associated with the inhibition of germination and seedling growth of the weeds *S. vulgaris* and *L. perenne*. Our laboratory findings suggest an allelopathic effect of bulb extracts. The two alkaloids may be excreted into the soil when bulbs are propagated or wounded to inhibit sprouting or growth of competing plants. It is known that lycorine is a major compound of the mucilage leached by *Narcissus* bulbs when wounded [23]. Alternatively, if present in the leaves, the two alkaloids may be released into the soil with decomposing plant material. The presence of narciclasine in the leaves of *N. pseudonarcissus* cv. Carlton has been shown by Lubbe *et al.* [20]. Concentrations of narciclasine in the leaves were double compared to those in the bulbs. Based on these results the leftover biomass from flower bulb production forms a promising source of allelochemicals for further studies on weed growth inhibition. The activity of narciclasine and lycorine on other problematic weeds, their activity and release from different plant parts under lab as well field-conditions and their potential application as extracts, cut-up pieces of bulbs exudating mucilage or as cover mulch form the basis of further studies.

## 3. Experimental

### 3.1. Plant Materials

All methanol and chloroform extracts used in this study were obtained from the extract library of Holland Biodiversity B.V. (Lisse, The Netherlands). The extracts were based on bulbs, leaves or flowers. Thirty three species deriving from nine families were studied as shown in Table 1.

### 3.2. Chemicals

Narciclasine (>98% pure) and lycorine hydrochloride (98% pure) were obtained from Sigma-Aldrich (Steinheim, Germany). The organic mobile phases for LC-MS and HPLC analysis were LC-MS grade and HPLC grade and were also obtained from Sigma-Aldrich. All organic solvents used for bulk extraction including partitioning, fractionation and preparation of standard solutions were analytical grade and purchased from Sigma-Aldrich. Deionised water was obtained from the Natural Product Laboratory, Institute of Biology, Leiden University.

### 3.3. Bioassays

The major weeds in Dutch flower bulb production comprise *Senecio vulgaris* (common groundsel), *Urtica urens* L. (annual nettle), *Persicaria maculosa* Gray (redshank), *Fallopia convolvulus* L. (black bindweed), *Rorippa sylvestris* L. Besser (yellow fieldcress), *Equisetum arvense* L. (field horsetail), *Stellaria media* (L.) Vill. (common chickweed), *Cyperus esculentus* L. (yellow nutsedge), *Elytrigia repens* L. (quackgrass), *Poa annua* L. (annual bluegrass) and *Polygonum aviculare* L. (common knotgrass). In this study, we used *S. vulgaris*, a major dicotyledoneous weed and *L. perenne*, a minor monocotyledonous weed in Dutch flower bulbs. The later has been originally used to develop the germination inhibition bioassay [9] we applied, adapted to a larger sample size.

#### 3.3.1. Germination Inhibition Bioassay

Each extract was tested at two concentrations, 1 mg/mL and 0.5 mg/mL. The methanol extracts were dissolved in an aqueous solution of 0.05% Tween 20. Tween 20 served as a surfactant and is known for its non-toxic effects on seedlings. The chloroform extracts were dissolved in methanol. Subsequently, the extracts were applied on filter paper (Whatman No. 1) fitting the wells of a 6 well plate and allowed to evaporate to dryness. The filter papers of the chloroform extracts were then moistened with 0.8 mL of a 0.05% (v/v) aqueous solution of Tween 20. The aqueous solution of 0.05% Tween 20 served as a negative control. Chlorpropham, a commercial herbicide, at a concentration of 0.23 mM, was used as a positive control. Ten seeds of the weed to be tested were placed on the filter papers and incubated at 25 °C (L:D 12:12) for 7 days at the end of which the number of germinated seeds was counted. Seeds were considered germinated if the emerging radicles were longer than 2 mm. Each treatment at each concentration was replicated three times. Differences in germination inhibition between the plant extracts and the control were analyzed by chi-square tests.

#### 3.3.2. Growth Inhibition Bioassay

Growth inhibition of germinated seeds was tested using the most promising extracts deriving from the germination inhibition bioassay, at lower concentrations. As such, 10 methanol extracts at concentrations of 0.1 mg/mL and 0.01 mg/mL were tested against *S. vulgaris*. The bioassay was performed as describe above. After 7 days of incubation at 25 °C the length of coleoptiles and radicles were measured. Each treatment at each concentration was replicated three times. Significant differences in coleoptile and radicle growth between treatments and control plants were analyzed by one-way ANOVA.

### 3.4. Identification and Purification of Active Compounds

#### 3.4.1. Narciclasine

The methanol extracts which at higher concentrations inhibited germination and at lower concentrations inhibited growth of *S. vulgaris*, were used for active compound identification using HPLC followed by LC-MS. HPLC was performed on an Agilent 1200 system equipped with an auto sampler, a quaternary pump and a photodiode array detector (Polymer Laboratories, Varian Inc.) operating at 230, 254 and 330 nm. For the UV-spectra reading, 230 nm was used to identify narciclasine. Thirty micro liters each of 1 mg/mL sample dissolved in methanol were injected. The samples were separated with a Phenomenex Luna C_18_-RP (250 × 4.60 mm, 5.0 µm) column at room temperature. The mobile phase consisted of deionised water with 0.1% formic acid and methanol with 0.1% formic acid. The gradient solvent of 20% methanol to 90% water in 30 min time was utilized with a flow rate of 0.5 mL/min. Data were recorded and analyzed using chemstation for LC 3D system software.

LC-MS was performed to obtain the mass spectra of the compounds detected in the HPLC analysis. LC-MS was performed on an Agilent 1100 series LC-MSD, equipped with an Agilent Eclipse XDB C_18_ (250 × 4.60 mm, 5.0 µm) column. The mobile system used was the same as described for HPLC above. The obtained UV- and mass spectra were then compared with the existing literature. In this way narciclasine, was identified as the active compound.

#### 3.4.2. Lycorine

A large scale extraction of *A. belladonna* was performed based on 1 kg *A. belladonna* bulbs obtained from Holland Biodiversity B.V. The bulbs were frozen with liquid nitrogen and ground with a Waring laboratory blender (Waring Products Inc., Torrington, CT, USA). The ground material was freeze-dried and stored at 4 °C until further workup. For extraction, 400 g of the freeze dried material was macerated with 750 mL 70% aqueous methanol for 20 min in an ultrasonic bath at room temperature. After filtration, the residue was extracted again as described. The two extracts were combined and the solvent was evaporated to dryness using a rotary evaporator at 50 °C.

Subsequently, the extract was fractionated using liquid-liquid partitioning with a separatory funnel using water, hexane, chloroform, and butanol as solvents. Thirty five gram of extract was dissolved in 500 mL of distilled water to which 500 mL of hexane was added and shaken thoroughly until the water and hexane phases were clearly separated. Partioning was performed twice. The two hexane fractions were combined and the solvent was removed by evaporation using a rotary evaporator at 70 °C. Subsequently, the water phase was partioned using 500 mL chloroform, followed by a further partition with 500 mL butanol. Each partition was conducted twice, the two solvent fractions were combined and evaporated with a rotary evaporator at 50 °C. Finally, the remaining water fraction was evaporated using a freeze-dryer.

The hexane and chloroform fractions did not provide sufficient material for further bioassays and purification analysis. Therefore, we focused on the butanol and water fractions, which were tested in an inhibition bioassay described above. In contrast to the water fraction the butanol fraction, showed a strong germination inhibition and was thus further fractionated using a sephadex column. Four gram of the butanol fraction was applied on a Sephadex LH-20 column, 1 cm in diameter and 25 cm in height. The column was eluted with methanol as an isocratic solvent system. Nine fractions of 3 mL each were collected based on their volume. The fractions were concentrated using a nitrogen dryer. Subsequently, the nine fractions obtained were tested in the inhibition bioassay described above. The highest inhibitory activity was observed in fractions 4 and 5 which thus were subjected to HPLC and LC-MS analysis for active compound identification. For HPLC the method by Ptak and Tanchy [39] with slight modifications of the gradient was applied. The mobile phase was eluted at a flow rate of 0.8 mL/min using a gradient ranging from 20% solvent A to 100% solvent B in a time of 20 min. Solvent A consisted of 97.5% of 10 mM NH_4_HCO_3_ with 2.5% of MeOH (pH 7.8) and solvent B consisted of 2.5% of 10 mM NH_4_HCO_3_ with of 97.5% MeOH. A Phenomenex Luna C_18_-RP (250 × 4.60 mm, 5.0 µm) column at room temperature was used. Measurements were taken at a wavelength of 254, 280, and 330 nm. For the UV-spectra reading, 280 nm was used to identify lycorine. One major peak was observed in fraction 4 and 5, which was further analyzed using LC-MS analysis as described above. Comparing the UV- and mass spectra with the existing literature the compound was identified as lycorine, which was confirmed using a standard reference. For quantification of lycorine, a standard solution was prepared by dissolving 1 mg of lycorine in 1 mL of methanol. The standard solution was injected into the HPLC at 0.10, 0.50, 2.50, 5.00 and 10.00 µL to construct a standard curve.

### 3.5. Growth Inhibition Activity of Narciclasine and Lycorine

Dose-response curves were obtained for concentration of 0.5, 0.25, 0.05, 0.025 and 0.005 µM narciclasine and 200, 130, 30, 3 and 0.3 µM lycorine. Each concentration was replicated three times. Applying probit analysis the concentration at which 50% of the *S. vulgaris* seed germination (IC_50_) was inhibited and its 95% confidences interval was calculated. In addition, the IC_50_ of the *S. vulgaris* seed germination of chlorpropham was determined at concentrations of 0.47, 0.23 and 0.047 mM.

## 4. Conclusions

In the present study we identified narciclasine and lycorine as potential allelochemcials in spring flower bulbs using bioassay-guided fractionation. Both alkaloids were associated with a strong inhibition of germination and seedling growth of the weeds *S. vulgaris* and *L. perenne*. As such, the leftover biomass from flower bulb production represents a promising source of allelochemicals for further studies on weed growth inhibition, including their activity on other problematic weeds, their mode of release, their affectivity in the field and potential methods of application.
